# Patients’ Experiences of Accessing Their Electronic Health Records: National Patient Survey in Sweden

**DOI:** 10.2196/jmir.9492

**Published:** 2018-11-01

**Authors:** Jonas Moll, Hanife Rexhepi, Åsa Cajander, Christiane Grünloh, Isto Huvila, Maria Hägglund, Gunilla Myreteg, Isabella Scandurra, Rose-Mharie Åhlfeldt

**Affiliations:** 1 Department of Information Technology Uppsala University Uppsala Sweden; 2 School of Informatics University of Skövde Skövde Sweden; 3 School of Electrical Engineering and Computer Science KTH Royal Institute of Technology Stockholm Sweden; 4 Institute of Informatics TH Köln University of Applied Sciences Gummersbach Germany; 5 Department of ALM Uppsala University Uppsala Sweden; 6 Department of Women's and Children's Health Uppsala University Uppsala Sweden; 7 Department of Business Studies Uppsala University Uppsala Sweden; 8 Centre for Empirical Research on Information Systems Örebro University School of Business Örebro Sweden

**Keywords:** eHealth, medical records, national survey, patients, patient-accessible electronic health records, patient portal, personal health records

## Abstract

**Background:**

Internationally, there is a movement toward providing patients a Web-based access to their electronic health records (EHRs). In Sweden, Region Uppsala was the first to introduce patient-accessible EHRs (PAEHRs) in 2012. By the summer of 2016, 17 of 21 county councils had given citizens Web-based access to their medical information. Studies on the effect of PAEHRs on the work environment of health care professionals have been conducted, but up until now, few extensive studies have been conducted regarding patients’ experiences of using PAEHRs in Sweden or Europe, more generally.

**Objective:**

The objective of our study was to investigate patients’ experiences of accessing their EHRs through the Swedish national patient portal. In this study, we have focused on describing user characteristics, usage, and attitudes toward the system.

**Methods:**

A national patient survey was designed, based on previous interview and survey studies with patients and health care professionals. Data were collected during a 5-month period in 2016. The survey was made available through the PAEHR system, called *Journalen*, in Sweden. The total number of patients that logged in and could access the survey during the study period was 423,141. In addition to descriptive statistics reporting response frequencies on Likert scale questions, Mann-Whitney tests, Kruskal-Wallis tests, and chi-square tests were used to compare answers between different county councils as well as between respondents working in health care and all other respondents.

**Results:**

Overall, 2587 users completed the survey with a response rate of 0.61% (2587/423,141). Two participants were excluded from the analysis because they had only received care in a county council that did not yet show any information in *Journalen*. The results showed that 62.97% (1629/2587) of respondents were women and 39.81% (1030/2587) were working or had been working in health care. In addition, 72.08% (1794/2489) of respondents used *Journalen* about once a month, and the main reason for use was to gain an overview of one’s health status. Furthermore, respondents reported that lab results were the most important information for them to access; 68.41% (1737/2539) of respondents wanted access to new information within a day, and 96.58% (2454/2541) of users reported that they are positive toward *Journalen*.

**Conclusions:**

In this study, respondents provided several important reasons for why they use *Journalen* and why it is important for them to be able to access information in this way—several related to patient empowerment, involvement, and security. Considering the overall positive attitude, PAEHRs seem to fill important needs for patients.

## Introduction

Internationally, there is movement toward providing patients with Web-based access to their electronic health records (EHRs); this parallels a global shift toward increased patient empowerment and patient participation. In the United States, for instance, the *OpenNotes* initiative for providing patients access to their EHR began as a pilot and evaluation project that included 105 volunteer primary care physicians and their 19,000 patients [1,2]. The project started in 2010 and has since spread throughout the United States [3]. *Blue Button* is a similar initiative by the United States Department of Veteran Affairs, which enables Veteran Affairs patients to access data from their EHR through Web, such as clinical notes, Veteran Affairs appointments, test results, etc [4,5]. Similar schemes have been initiated in Australia [6], Finland [7], Canada [8], Denmark [9], Estonia [10], the United Kingdom [11], and Sweden [12]. However, different strategies and approaches have affected the uptake and impact. The implementation progress has in several countries been slow because of legal constraints [13,14] and concerns about, for example, security and privacy among medical professionals [8,12,15].

In Sweden, citizens are provided with the service *Journalen* for patient-accessible EHR (PAEHR), accessible through Web via the national patient portal. The PAEHR service accesses the EHR information through a national health information exchange platform. Hence, patients have one access point to all their health record information regardless of (1) how many health care providers they have visited, and (2) which EHR system their health care providers use [16]. However, there are limitations and exceptions to patient access. Whether patients have access to their EHR depends on whether they receive care from a public or private health care provider. For example, it is possible that although the county has implemented *Journalen*, specific private providers do not give access to their notes. At the time of the study, each health authority could choose whether they give patients immediate access to signed (ie, confirmed) notes, unsigned notes, or whether a delay of 14 days is implemented to either or both of them [17,18]. There seems to be no standard practice for physicians to sign notes [19] and some notes are never signed [20]. Following a court decision that deemed health care notes in the records as “public documents” and, thus, patients having the right to read them, the implementation in Region Uppsala was changed to let patients decide what kind of notes they want to read [20].

**Figure 1:**
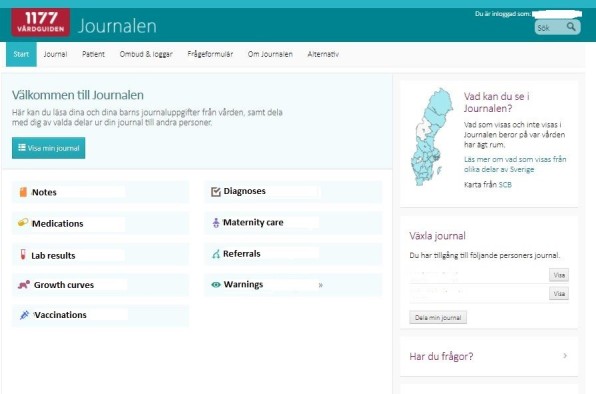
The patient-accessible electronic health record *Journalen* after log-in, showing the functions and information available (partially translated). Licensed under fair use. Source: https://e-tjanster.1177.se/. Service produced by Inera AB under the auspices of Swedish county councils and regions.

**Figure 2:**
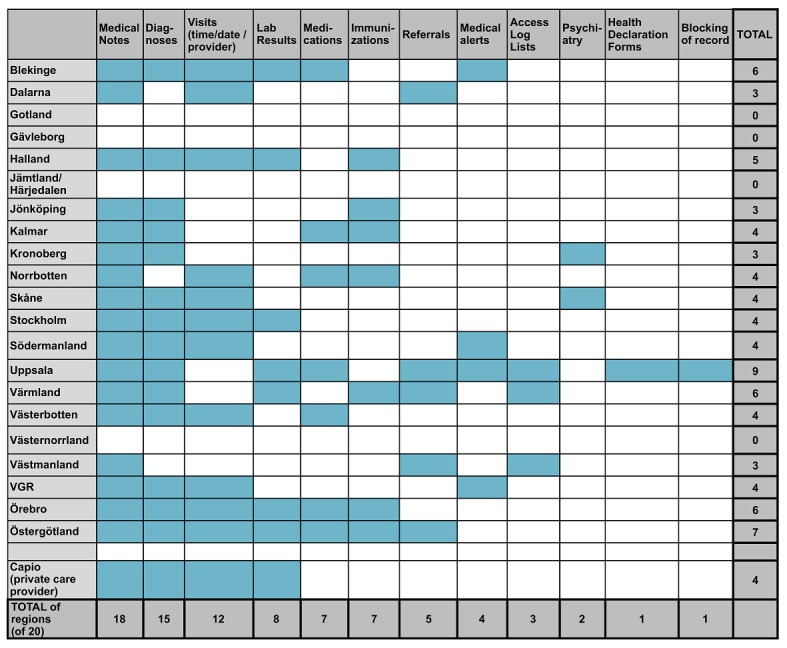
Information shown in Journalen depending on county council or health care provider (blue squares) during the time of the survey.

Currently, when patients access the PAEHR, they find varying clinical content, such as medical notes from the EHRs (from all health care professions and all connected health care providers who have agreed to give access, both public and private), a list of prescribed medications, lab results, warnings, diagnosis, maternity care records, referrals, and vaccinations (Figure 1). Although the PAEHR interface is identical for all users, there are significant differences in how much information to which each health care provider gives access. Figure 2 gives an overview of what types of clinical content the health care providers have chosen to give access to. The *access log list* (also called “consult audit trail”) presents a list of everyone who has accessed the record [13], including their name, role, and the date they accessed the record. The list includes health care professionals as well as anyone patients have chosen to share their record with, and patients can make use of the list to check whether there has been unexpected access.

Although patients’ use of and attitudes toward PAEHRs have been studied to some degree, research on PAEHRs has up until today mostly focused on the health care professionals’ perspective [8,12,19,21,23], and most studies originate from the US context [23]. For example, health care professionals have had several concerns such as the negative impact on workload, privacy risks, and fear of increased anxiety in patients [23]. However, in contrast to the fears of many health care professionals, a Swedish interview study with cancer patients showed that Web-based access did not generate substantial anxiety, concerns, or increase in the number of telephone contacts to care units [22]. The same study showed that the patients were generally positive toward the system and the possibilities it gives to read and follow their medical treatment. In addition, an extensive meta-study by Mold et al [24] on patients’ attitudes showed a similar generally positive attitude. Improved communication with clinicians, as well as improved satisfaction and self-care, were among the most important findings. Similar conclusions were drawn in a meta-study by de Lusignan et al [23], indicating that Web-based access to own EHR and related services offers increased convenience and satisfaction to patients. A review published in 2015 called for more empirical testing regarding the effect of PAEHR on outcomes for both patients and health care providers [25]; this is especially true for follow-up studies, investigating the effects of long-term use of PAEHR systems. In addition, the meta-study by Mold et al [24] concluded that most studies on PAEHR focusing on patients’ usage and attitudes had been conducted in relation to the *OpenNotes* movement in the United States. To date, few follow-up studies have been conducted in Europe and none in Sweden. The Swedish interview study with patients performed by Rexhepi et al [22] was only concerned with patients with cancer in Region Uppsala and was conducted shortly after *Journalen* was introduced. Hence, it did not answer questions about which types of patients used the Swedish PAEHR system or attitudes toward the system in the wider population representing patients with other conditions and from different county councils with different implementations. The large Swedish national survey study about PAEHR use and attitudes presented in this paper aims to fill the abovementioned research gaps by answering the following research questions several years after *Journalen* was introduced:

- Why do patients in Sweden use *Journalen*? And how often do they use it?
- What types of information are most valued by patients?
- What are the general attitudes by patients toward *Journalen*?
- What differences can be identified with regards to attitudes between different county councils in Sweden?

After the survey study has been introduced in section 2, results regarding demographics, usage, and attitudes toward *Journalen* will be presented in section 3. Section 4 includes discussions of key results and their relations to earlier studies with patients as well as health care professionals. The paper ends with conclusions and a short discussion about the need for further studies in this area.

## Methods

A survey study was conducted to elicit opinions and experiences of patients using *Journalen*. The study was conducted from June to October 2016, after ethical approval of the research was granted by the Regional Ethical Review Board in Uppsala, Sweden (EPN 2016/129). Participants were recruited through the national electronic health (eHealth) service *Journalen*. When patients logged into the service *Journalen*, they received a request for voluntary survey participation together with information about the study. Thus, only active users of *Journalen* received a request for participation. Patients were automatically presented with standard consent on Web prior to completing the survey. Participants accessed the survey, and the possibility to give consent, by following a link beneath the information about the study.

An anonymous self-completion questionnaire was designed covering six different topic areas with a total of 24 questions (see questionnaire in Multimedia Appendix 1):

- General questions related to the eHealth service *Journalen*
- Questions targeting experiences from accessing and using the content of *Journalen*
- Information security
- General questions about information needs, behavior, and information-seeking styles
- Personal health-related questions
- Demographics

The questionnaire included questions with various response options (5-point Likert scale, multiple choice, and free text form). The selection of topic areas and the design of the questionnaire was informed by previous studies, including Huvila et al [26], Rexhepi et al [22], Grünloh et al [19], Huvila et al [27], Ålander & Scandurra [28], and Scandurra et al [21]. The usability and technical functionality of the electronic questionnaire had not been tested before fielding the questionnaire. However, participants received information about whom to contact in case of technical issues. Overall, 2587 patients from 21 county councils completed the survey. The number of respondents for each county council or region varied. Notably, it was not possible to statistically verify whether the number of respondents is adequate to provide other than some tentative county and group-wise comparisons.

The collected data were managed by the eHealth service provider Inera in accordance with the security requirements presented in the ethical application and approved from the Regional Ethical Review Board. The survey data were stored in the same database system as the PAEHR, meaning that the collected data, including patient identification (ID), had the same security protection as all patient information handled in the PAEHR. A patient ID was stored during the collection period to ensure that patients have not left duplicate responses. When the collection period was completed, the patient ID was removed and all stored information was anonymized. After being collected, the anonymized dataset was exported to researchers for analysis. In order to provide a clear focus on the research questions on which this study is based, a selection of themes and questions were made. Questions regarding demography, general usage data, and attitudes were selected for analysis. Before the analysis began, data from patients only treated in Region Gävleborg were excluded (2 patients) because *Journalen* had not yet been introduced in that county council at the time the survey was open. It was, however, possible to access the e-service and the survey without having access to any EHR data, and as these respondents had only received care in that county council, their experiences were considered not relevant for this study.

The results presented in this paper are based on the descriptive analysis showing general trends related to the themes mentioned above. The reported percentages are based on those who answered each specific question—the total number of respondents on each question are indicated either in the main text or Multimedia Appendix 2. Only completed questionnaires have been analyzed, as the answers were stored in the database only when the respondent chose to submit on the last page. The Mann-Whitney U test was used for Likert scale items after a transformation (strongly agree=5; strongly disagree=1) for pairwise comparisons between the group of respondents who worked or had worked in health care and the group consisting of all other respondents. In addition, chi-square test was used for questions with nominal scales. The Kruskal-Wallis test was used to detect possible differences in ratings between county councils. The significance level was set to 95% for all tests. The SPSS 25 software was used for all analyses.

## Results

### Result Presentation

Results regarding the users of *Journalen*, as well as their usage of the system and attitudes toward it, are presented below. References to specific questions in the appended questionnaire will be given as *{qX}*, where *X* denotes the question number.

### Demographic Information

During the survey period, 423,141 users logged into *Journalen*, of which 2587 patients completed the survey (unique users that logged in; response rate, 0.61%, 2587/423,141). Although not everyone answered all the questions, >90% (2338/2587) responded to each question in the survey. Of all respondents, 62.97% (1629/2587) identified as women and 30.85% (798/2587) as men; 0.39% (10/2587) respondents chose “other” and 150 did not answer this question. According to the statistics about *Journalen* from the company providing the service, Inera [29], this reflects the gender distribution of the users in general (in 2016: 60% women and 40% men). Of all respondents, 39.81% (1030/2587) stated that they were working or had been working within health care and 54.54% (1411/2587) stated that they had no professional relation to health care (146 respondents did not answer this question). Independent of the county council or region a person lives, it is possible to receive care in a different county as well. In addition, 1674 respondents indicated that they, indeed, had received care from a county council other than their home county council. Table 1 shows from which county council or region respondents come from, as well as the number of respondents that received care in the respective council or region. Of note, county councils, which had not yet introduced *Journalen* at the time when the survey was closed are not included in the third column of Table 1. Table 2 shows the educational levels represented among respondents. Respondents with at least 3 years of higher education background are in the majority.

To sum up, the survey results regarding user characteristics on a national level indicate that a majority of respondents were women and that the majority had studied at least 3 years on the higher education level. In addition, results indicate that many users of *Journalen* had experiences both of being patients and working as medical professionals.

**Table 1:** The number of participating patients from each county council or region and the total number of respondents who have received care in the respective county council or region.

County council or region	Respondents from respective county council or region, n	Respondents who received care in the respective county council or region, n
Region Skåne	520	692
Region Uppsala	333	520
Region Östergötland	241	364
Region Västra Götaland	179	328
Region Jönköping	154	218
Värmland county council	143	180
Västmanland county council	103	163
Region Örebro	102	185
Sörmland county council	94	149
Region Kronoberg	94	133
Dalarna county council	93	160
Västerbotten county council	93	144
Kalmar county council	78	133
Norrbotten county council	57	98
Region Halland	54	106
Blekinge county council	51	101
Stockholm county council	41	299
Region Gävleborg	7	N/A^a^
Västernorrland county council	6	N/A
Region Gotland	1	N/A
Region Jämtland Härjedalen	1	N/A
Not specified	142	N/A

^a^N/A: not applicable.

**Table 2:** Educational level of persons who answered the survey (N=2455).

Educational level	Value, n (%)
Research education	75 (3.05)
Higher education ≥3 years	945 (38.49)
Higher education <3 years	467 (19.02)
High school ≥3 years	410 (16.70)
High school <3 years	248 (10.10)
Less than high school	159 (6.48)
No formal education	66 (2.69)
Other	85 (3.46)

### Usage

Regarding the frequency of use *{q2}*, 72.08% (1794/2489) of the patients answering the survey logged into *Journalen* about once a month, whereas others logged in about once a week (240/2489, 9.64%), a few times a week (393/2489, 15.79%), and several times a day (62/2489, 2.49%). Thus, the majority of patients answering the survey were infrequent users. The chi-square test showed no statistically significant association between respondents who were working or had been working in health care and those who had not (*χ*^2^_4_=1.5, *P*=.83).

Table 3 shows a mapping between the frequency of use data and self-reported demographic data about health conditions *{q23, q24}*. Persons who considered their health as “very good” were among the least frequent users, whereas those with cancer, diabetes, or other chronic conditions were among the most frequent users. Many health care providers chose not to give access to psychiatric records, yet respondents who stated that they have a psychiatric condition appeared to access the record similarly to the aforementioned conditions. They may, of course, have other health issues that they are interested in but based on these results, there appears to be no major difference in how patients who identify as belonging to psychiatry access their records.

**Figure 3:**
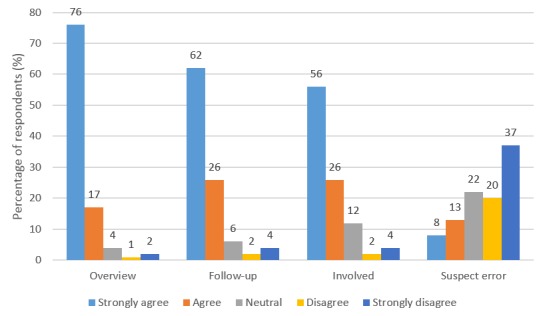
Percentage of respondents who strongly agree, agree, are neutral, disagree, or strongly disagree with the statement that they use Journalen to get an overview of their medical history and treatment, to follow up on what has been said during visits, to become more involved in their own care, and because they suspect inaccuracies, respectively *{q4}*.

One part of the patient survey focused on why they were using *Journalen {q4}*. The three most common reasons were gaining an overview, following up on visits, and becoming more involved, and the least common reason was that they suspected inaccuracies (Figure 3). The most common reason for using *Journalen* was to obtain an overview of one’s medical history and treatment. Multimedia Appendix 2 provides more detailed results from all items in *{q4}*.

Mann-Whitney *U* test showed no statistically significant difference between the group of current or former health care professionals and the group of all other respondents regarding the reasons *overview* (*U*=717,488, *P*=.13), *follow-up* (*U*=699,543, *P*=.83), *become more involved* (*U*=655,657, *P*=.16), and *suspect error* (*U*=653,566, *P*=.50). Significant differences between these groups were only found for *not being sure about treatment* (*U*=704,309, *P*=.001) and *prepare for a visit* (*U*=714,054, *P*=.003). In both these cases, current and former health care professionals gave significantly lower ratings. See detailed results in Multimedia Appendix 2.

When asked about how long respondents were willing to wait until information was available after a visit *{q6}*, 68.41% (1737/2539) wanted access to new information same day or after a day (in other words, within 24 hours). Furthermore, 19.22% (488/2539) respondents wanted access to new information within 2 weeks, 1.42% (36/2539) within 1 month, and 10.95% (278/2539) chose “other.” Respondents were informed that the alternatives “Same day” and “After a day” would mean that the physician may not yet have signed the notes and probably not have seen, for instance, new test results. The chi-square test showed no statistically significant association between respondents who were working or had been working in health care and those who had not (*χ*^2^_5_=7.8, *P*=.17).

To sum up, the survey results showed that most respondents were infrequent users of *Journalen*, which was especially true for users who considered themselves to be in good health. Patients with chronic conditions were among the most frequent users. The main reasons for using *Journalen* were (1) to receive an overview of one’s own medical history and treatment; (2) to follow up on doctor’s visits; and (3) to become more involved in one’s care.

### User Attitudes and Perceptions

Overall, patients who answered the survey were positive toward *Journalen*, as indicated in Table 4. Of all respondents, >96% (2455/2528 and 2454/2541 for the respective questions presented in Table 4) showed a positive attitude (strongly agree or agree) toward *Journalen {q3}*. As can be seen in Table 5, there are no substantial differences between county councils regarding attitudes toward the reform. In both Tables 4 and 5 the responses “Strongly agree” and “Agree” have been summed up as “Positive” and “Strongly disagree” and “Disagree” have been summed up as “Negative.” A Kruskal-Wallis test showed no statistical differences in attitude ratings between the different county councils (*χ*^2^_15_=10.7, *P*=.77), showing no support for an effect of the county council. The most positive respondents (98.04%, 150/153, positive and 0.65%, 1/153, negative) belonged to the Dalarna county council, and the least positive (93.62%, 88/94, positive) respondents belonged to Blekinge county council. More detailed results from *{q3}*, for the respective county council, can be found in Multimedia Appendix 2.

Statistically significant differences between the group of current and former health care professionals and the group of all other respondents were found both regarding *Journalen* as a reform (*U*=784,071, *P*<.001) and *Journalen* as good for them (*U*=728,196, *P*=.005). In both these cases, current or former health care professionals were significantly more negative in their answers. See detailed results for these two groups in Multimedia Appendix 2.

Owing to large differences between county councils regarding what functions were available in *Journalen*, it is important to consider the county council when evaluating user attitudes toward and perceived importance of *{q17}* different functions. In Table 6, answers about the perceived importance from respondents who belonged to county councils or regions where a certain type of information was shown are compared with the answers from respondents from county councils or regions where the information was not shown. Overall, access to test results is perceived to be the most important category and log list the least important. More detailed results from the chosen items in *{q17}*, for the respective county council, can be found in Multimedia Appendix 2.

When comparing current and former health care professionals against the group of all other respondents for the information types presented in Table 6, significant differences were found for immunizations (*U*=640,633, *P*<.001), health declaration forms (*U*=615,435, *P*<.001), and log list (*U*=640,253, *P*=.004). In all these cases, current or former health care professionals gave significantly higher ratings. See detailed results in Multimedia Appendix 2. Statistically significant differences in ratings between county councils or regions that present a certain type of information and those who do not were only found for test results (*U*=710,736, *P*=.049) and visit history (*U*=632,152, *P*=.005). Thus, whether or not the information is accessible in a particular county council does not appear to have a significant impact on the rating of importance of the most of the information types.

**Table 3:** Frequencies of usage of *Journalen* by respondents belonging to different condition categories.

Condition	Several times a day, n (%)	Few times a week, n (%)	Once a week, n (%)	About once a month, n (%)
Cancer (n=335)	18 (5.4)	70 (20.9)	40 (11.9)	207 (61.8)
Diabetes (n=260)	6 (2.3)	50 (19.2)	35 (13.5)	169 (65.0)
High blood pressure (n=589)	17 (2.9)	84 (14.3)	60 (10.2)	428 (72.7)
Psychiatry (n=487)	11 (2.3)	98 (20.1)	48 (9.9)	330 (67.8)
In good health (n=1096)	22 (2.0)	114 (10.4)	86 (7.8)	874 (79.7)

**Table 4:** Number of respondents who considered access to *Journalen* as good for them (N=2528) and as a good reform in general (N=2541) *{q3}*.

Question	Positive, n (%)	Neutral, n (%)	Negative, n (%)
“Getting access to *Journalen* is good for me”	2455 (97.11)	39 (1.54)	34 (1.34)
“Allowing access to *Journalen* is generally a good reform”	2454 (96.58)	38 (1.50)	49 (1.93)

**Table 5:** Number of participants from different county councils who were positive, neutral, and negative toward *Journalen* as a reform within health care.

County council	Positive, n (%)	Neutral, n (%)	Negative, n (%)
Region Skåne (n=653)	636 (97.3)	6 (1.0)	11 (1.7)
Region Uppsala (n=491)	477 (97.1)	6 (1.2)	8 (1.6)
Region Östergötland (n=341)	327 (95.9)	6 (1.8)	8 (2.3)
Region Västra Götaland (n=304)	286 (94.1)	8 (2.6)	10 (3.3)
Stockholm county council (n=291)	278 (95.5)	5 (1.7)	8 (2.8)
Region Jönköping (n=203)	199 (98.0)	2 (1.0)	2 (1.0)
Region Örebro (n=169)	162 (95.9)	6 (3.5)	1 (0.6)
Värmland county council (n=170)	163 (95.9)	4 (2.3)	3 (1.8)
Västmanland county council (n=153)	148 (96.7)	3 (2.0)	2 (1.3)
Dalarna county council (n=153)	150 (98.0)	2 (1.3)	1 (0.7)
Sörmland county council (n=138)	134 (97.1)	2 (1.4)	2 (1.4)
Västerbotten county council (n=139)	132 (95.0)	2 (1.4)	5 (3.6)
Region Kronoberg (n=128)	123 (96.1)	2 (1.6)	3 (2.3)
Kalmar county council (n=128)	123 (96.1)	2 (1.6)	3 (2.3)
Region Halland (n=101)	96 (95.0)	3 (3.0)	2 (2.0)
Blekinge county council (n=94)	88 (93.6)	3 (3.2)	3 (3.2)
Norrbotten county council (n=94)	92 (97.9)	0 (0.0)	2 (2.1)

**Figure 4:**
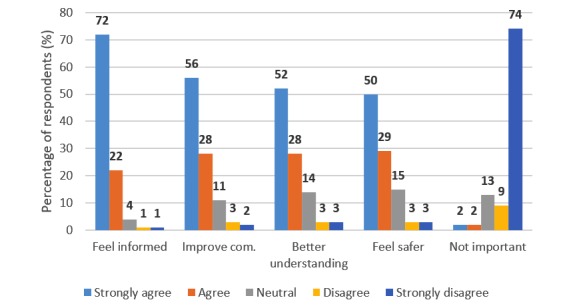
Percentage of respondents who strongly agree, agree, are neutral, disagree, or strongly disagree with that accessing patient information is important for them because it makes them feel informed, improves communication between medical staff and them, improves the understanding of their condition, or makes them feel safe; the “Not important” option is shown as a comparison *{q5}*.

When asked about the importance of being able to access patient-related data, >93% (2348/2506) of respondents strongly agreed or agreed with the statement that it made them feel more informed (Figure 4). The diagram shows the four highest rated reasons to why respondents believed the access to patient-related information to be of importance to them *{q5}.* These are as follows: (1) it makes them feel informed; (2) it improves communication between medical staff and them; (3) it improves the understanding of their condition; and (4) it makes them feel safe. As a comparison, the results for the alternative “Not important” are also provided in Figure 4. It is clear that the majority of respondents found value in being able to access information about their health. More detailed results from all items in *{q5}* can be found in Multimedia Appendix 2.

Several significant differences were found when using the Mann-Whitney U test to compare ratings from current and former health care professionals with all other respondents. Significant differences were found regarding the items feel informed (*U*=725,164, *P*=.02), improve communication (*U*=763,062, *P*<.001), better understanding (*U*=746,053, *P*<.001), and not important (*U*=585,143, *P*=.02). In all these cases, those who did not belong to the group of current or former health care professionals gave significantly more positive ratings. More details can be found in Multimedia Appendix 2.

Finally, 26.07% (655/2512) of respondents stated that they had felt worried about something they had read in *Journalen {q12}*. Thus, the majority of respondents have not been worried by the contents of *Journalen*. The chi-square test did not show any significant association between the group of current or former health care professionals and the group of all other respondents (*χ*^2^_2_=2.9, *P*=.24).

When asked about what actions they took in cases when they felt worried after reading in *Journalen*, the most common answer of the respondents was that they had searched for information on the internet (339/655), followed by calling the hospital (237/655) and asking during the next doctor’s visit (235/655). The least common action to take was to contact a patient association (36/655).

To sum up, the survey results revealed a strong positive attitude toward *Journalen* as a reform and a resource. The top three reasons why patients believed that *Journalen* is important were as follows: (1) it makes them feel more informed; (2) it improves their communication with medical staff; and (3) it results in a better understanding of one’s health status. The most important resource, according to the survey, was test results.

**Table 6:** Comparisons between answers from county councils that present a particular type of information (Yes) and those who do not (No).

Information types		Value, n (%)		Mean (SD)	*P* value
**Referral**					.62
	Yes		894 (37.33)	4.60 (0.75)	—^a^
	No		1501 (62.67)	4.59 (0.79)	—
**Medlist**					.53
	Yes		933 (38.88)	4.61 (0.79)	—
	No		1467 (61.12)	4.61 (0.83)	—
**Immunization**					.88
	Yes		809 (33.96)	4.39 (0.94)	—
	No		1573 (66.04)	4.37 (1.02)	—
**Labresult**					.049
	Yes		948 (39.45)	4.75 (0.61)	—
	No		1455 (60.55)	4.77 (0.66)	—
**Visithistory**					.005
	Yes		1489 (62.43)	4.64 (0.75)	—
	No		896 (37.57)	4.57 (0.80)	—
**Healthforms**					.37
	Yes		560 (23.18)	4.14 (1.21)	—
	No		1856 (76.82)	4.09 (1.24)	—
**Loglist**					.26
	Yes		320 (13.22)	4.08 (1.04)	—
	No		2101 (86.78)	4.12 (1.07)	—

^a^Not applicable.

## Discussion

### Principal Results

Regarding attitudes, the main finding is that, similarly to earlier research, respondents are generally positive toward the service; this is congruent with findings related to other PAEHR studies, such as *Blue Button* [5] or *OpenNotes* [30]. Furthermore, attitudes do not differ greatly between patients from different county councils in Sweden; this shows that large differences between county councils, regarding which type of information is accessible, do not have a major effect on the overall acceptance of the service. The same tendency can be observed when considering the importance of having access to the different types of information (eg, test results) or the availability of a function (eg, ability to provide a health declaration for the next visit). Whether this function or information is currently available in that county or not does not to a large extent affect the rating of its importance. Statistical differences between county councils that provide a particular type of information and those who do not were only found for test results and health declaration forms; this result was expected, as the question regarded the information types as such and not their possible implementations in *Journalen*. In addition, it indicates that the implementations of the information sources in *Journalen* do not have a negative impact in this respect. According to Table 6, test results are perceived to be the most important information source. This is interesting because currently most county councils in Sweden do not show this information. These results give indications about which information types to prioritize in future development iterations. Regarding the perceived effects of using *Journalen*, the alternatives “Feeling informed” and “Improved communication with medical staff” are the most highly rated selected by respondents, which is supported by earlier findings from interviews with patients conducted right after launch in Region Uppsala [22].

When it comes to the usage, it is clear that the majority of respondents are not frequent users of *Journalen* (use frequency about once a month); this may be explained by that the majority of respondents indicated that they are in good health. Logging into *Journalen* frequently would not be relevant if an individual is currently not having an ongoing health issue with an active health care contact. Table 3 also shows that nearly 80% (874/1096) of those respondents answered that they are not frequent users. We do, however, know little about the patterns of use in relation to contact with health care and other health-related events; it is a topic to be explored further. In addition, Table 3 reveals that respondents with certain conditions, such as cancer or a psychiatric condition, belong to the more frequent users. The highest rated reasons for using *Journalen* among respondents (getting an overview, follow up on visits, and becoming more involved in the care process) correspond with results from earlier interviews with patients in Sweden [22], as well as studies from other countries [11,30,31]. It is clear that searching for errors in the record is not the main reason to use the service and that most patients who answered the survey have not been worried of something they have read in *Journalen*; this is also in line with earlier findings [22]. However, it is in contrast to the results from earlier interviews with physicians [11,19,28], who expressed concerns that patients will be worried when they read their records, or that they will start looking for errors and, hence, believed that PAEHR is not useful for patients. The fact that most respondents want to have information in *Journalen* within a day (thus potentially unsigned information) is also interesting to relate to the concern of physicians that patients will have access to results before the professionals do. Only around 26% (655/2512) of respondents have felt worried because of something they have read in *Journalen* and, thus, the results indicate that early access to, for instance, test results may not be a big issue in this respect. Further analysis of the relationship between immediate access and worry is, however, needed.

Most county councils where respondents have received care are represented by, at least, 250 respondents and only one of these councils is represented by <100 respondents (Norrbotten county council, where only 98 respondents had received care). This is important, as it shows that there are adequate data that make it meaningful to study each county council separately. Moreover, respondents of this survey seem to be quite mobile in their care seeking, and a total of 1674 positive responses were given to the question whether health care had been received outside respondents’ home county. The result of this mobility can, for instance, be that a patient can see results from lab tests performed in one council but not another. This mobility highlights the importance of having one national PAEHR service giving patients access to information from many different health care providers in one aggregated view. It does, however, also highlight the need for a more unified national regulatory framework, ensuring a streamlined information provision across health care providers in all county councils [17].

### Limitations

There are some limitations to this study. First, the link to the survey was available on *Journalen* ’s log-in page. Thus, only persons who have logged in or, at least, visited the log-in page to *Journalen* during the time that the survey was open are among respondents; this creates a positive bias, as previous users who no longer use *Journalen* for whatever reason have not answered the survey. This could, in part, explain the overwhelmingly positive overall attitudes toward the system. Another explanation for the overall positive attitudes could be that patients really are positive. There has been a lot of negative coverage on *Journalen* in Swedish media. Health care professionals, especially physicians, were critical to the service. Patients who are aware of this debate might have been reluctant to criticize the service out of fear of losing access altogether and rather express their support to ensure that the service continues. Either way, the results can only be interpreted as strong support of the service from respondents.

The results regarding the frequency of use show that most users log in a few times a month; this may be because most of the respondents do not have regular contact with health care. It is important to keep in mind that this question does not capture irregularities of use—users of *Journalen* probably log in more frequently when they are treated for an illness. In addition, it is important to consider that 1030 of 2441 respondents stated that they work or have worked within health care. It has been shown in earlier studies that, for example, physicians who had used *Journalen* themselves were more positive toward the service than those who had not [20,28]. The answers given by current and former health care professionals and all other respondents, respectively, have, however, been separated in Multimedia Appendix 2 and were similar for most questions used in the paper. Additionally, the Mann-Whitney tests did, for most items, not show any support for significant differences between these groups. The exception being the items in question 5 about why it is important to be able to access patient information.

Like in most surveys, respondents form a small sample of all possible users. A lot more users than those who answered the survey logged into *Journalen* during the 5 months the survey was open. Technically, 2587 respondents should be considered a good sample size; nevertheless, we do not know whether, for example, the demographic distribution is representative. Respondents have a higher education level than the general population. Among our respondents, 61% had higher education, whereas 42% of the general population does. Whether this is because users of *Journalen* are well-educated or whether this is a subgroup of users who are more inclined to answer a survey we cannot tell. Therefore, further studies of users of *Journalen* are needed.

### Conclusions and Future Work

Up until now, no study has investigated the long-term effects of PAEHRs in Sweden, and few follow-up studies on PAEHRs have been conducted in other European countries. In this paper, the much-needed follow-up work begins with a focus on patients and their usage preferences and attitudes toward the system. The results show that the majority of respondents are women, which is in line with the overall statistics on *Journalen*. Use frequency varies among patients with different conditions—the results indicate that patients with chronic conditions are among the most frequent users. In addition, results show that there is an overall strong positive attitude toward *Journalen* and no statistical differences in attitude ratings could be found between county councils. Thus, the difference in the information shown in *Journalen* between county councils does not have a strong effect in this respect. The highest rated reasons for using *Journalen* are to acquire an overview of one's health status and follow up on visits (eg, memory aid). Furthermore, results show that respondents view test results as the most important type of information, underlining the need to implement support for that information on a national level.

Further research is needed as there are many aspects of the patient survey which are outside the scope of this overview paper, like privacy and security, means of sharing information, usability, etc. In addition, within the recently started research project patient-centered assessment of patients’ online access to EHRs, the current implementation and use of PAEHR in Sweden will be evaluated through in-depth qualitative case studies in different regions to achieve a better understanding of how roles, relationships, and organizational structures are affected on micro, meso, and macro levels [16]. Furthermore, it is of importance to study the long-term effects that *Journalen* has had on the work environment for health care professionals, as this is also an area that is highly under investigated, especially in Europe.

## Appendix

Multimedia Appendix 1 The questionnaire including information about the study.

Multimedia Appendix 2 Detailed results from survey questions 3, 4, 5, and 17.

## Abbreviations

eHealth: electronic health
EHR: electronic health record
ID: identification
PAEHR: patient-accessible electronic health record
