# Supplementary material for: Patients’ Experiences of Accessing Their Electronic Health Records: National Patient Survey in Sweden
Source: J Med Internet Res. 2018 Nov 1;20(11):e278. doi: 10.2196/jmir.9492 (PMC6238103; doi:10.2196/jmir.9492)
Supplement: Multimedia Appendix 1 [file jmir_v20i11e278_app1.pdf]

Questionnaire - Patient empowerment
Evaluation of the patient's online medical records

The purpose of this survey is to identify and understand why patients choose to read their medical records online, how they experience the eHealth service and which other eHealth and information sources you would like to have access to in the future.

The survey provides a basis for the evaluation of e-health service “Journalen” and provide opportunities for you to influence the improvement of the eHealth service. Your feedback is important as it can help in developing better eHealth services in the future. The survey is part of research on eHealth performed by a group of researchers from Uppsala University, Örebro University, Lund University and the University of Skövde. The research is coordinated by Åsa Cajander (Uppsala University).

The results will be reported publicly, and be the basis for scientific articles.

We are happy to answer questions regarding the research and survey. For questions regarding the questionnaire study, contact

Rose-Mharie Åhlfeldt
rose-mharie.ahlfeldt@his.se
0730 - 67 72 67

For questions about the research project, please contact project manager

Åsa Cajander
asa.cajander@it.uu.se
0704-425 07 86

All responses are anonymous and thus cannot be traced to the informant. The data will not be transferred to third parties or used for commercial purposes.

Your participation is completely voluntary and you can cancel at any time the survey

General Questions on *Journalen*

1. I received information about the possibility to use *Journalen* via:

Medical professionals ☐
Media ☐
Information on the care unit ☐
Friends / Relatives ☐

Other (specify): ______________________________

2. How often do you use *Journalen*?

Several times a day ☐
Once a week ☐
Several times a week ☐
Once a month ☐

3. What is your attitude towards *Journalen*?

	Strongly Disagree	*Disagree*	*Neutral*	*Agree*	*Strongly Agree*
I believe that access to medical records online is generally a good reform	☐	☐	☐	☐	☐
I believe that access to *Journalen* is good for me	☐	☐	☐	☐	☐
I would consider to change healthcare providers to get one that gives me access to *Journalen*	☐	☐	☐	☐	☐

4. Why do you use *Journalen*?

	Strongly Disagree	*Disagree*	*Neutral*	*Agree*	*Strongly Agree*
Mostly general interest	☐	☐	☐	☐	☐
To get an overview of my medical history and treatment	☐	☐	☐	☐	☐
To get an overview of my relatives’ medical history and treatment	☐	☐	☐	☐	☐
Because I am not sure if I got the right care	☐	☐	☐	☐	☐
To follow up what has been said during a healthcare visit	☐	☐	☐	☐	☐
Because I suspect inaccuracies	☐	☐	☐	☐	☐
To prepare for my healthcare visit	☐	☐	☐	☐	☐
To become more involved in my care	☐	☐	☐	☐	☐

Other (specify): ______________________________

5. How important is it for you to be able to access patient information?

	Strongly Disagree	*Disagree*	*Neutral*	*Agree*	*Strongly Agree*
It improves communication between medical staff and me	☐	☐	☐	☐	☐
It leads to improvements in health and social care	☐	☐	☐	☐	☐
It improves the understanding of the condition	☐	☐	☐	☐	☐
It makes me feel safe	☐	☐	☐	☐	☐
It makes me feel informed	☐	☐	☐	☐	☐
It leads to that I can take care of my health better	☐	☐	☐	☐	☐
It leads to that I can take care of my relatives' health better	☐	☐	☐	☐	☐
It is essential that I am able to actively participate in decisions about my or my relatives' health	☐	☐	☐	☐	☐
For my own documentation	☐	☐	☐	☐	☐
It has no relevance	☐	☐	☐	☐	☐

Other (specify): ______________________________

6. How long do you think is reasonable to have to wait after a healthcare visit before you have access to your medical records via *Journalen*?

Same day (unsigned / uncertified information)	☐
After a day (unsigned / uncertified information)	☐
Two weeks (can be both signed / certified and unsigned / uncertified data)	☐
One month (can be both signed / certified and unsigned / uncertified data)	☐

Other (specify): ______________________________

7. To what extend do you agree with the following statements regarding your relationship with healthcare?

	Strongly Disagree	*Disagree*	*Neutral*	*Agree*	*Strongly Agree*
To take part of the patient information via *Journalen* has affected the relationship with healthcare system positively	☐	☐	☐	☐	☐
Medical staff has informed me about the possibility to read *Journalen*	☐	☐	☐	☐	☐
Medical staff has encouraged me to use *Journalen*	☐	☐	☐	☐	☐
I discuss the content of *Journalen* with medical staff	☐	☐	☐	☐	☐

8. What do you think about the usability of *Journalen*?

	Strongly Disagree	*Disagree*	*Neutral*	*Agree*	*Strongly Agree*
I think I want to use *Journalen* regularly	☐	☐	☐	☐	☐
I think that *Journalen* is more complicated than it needs to be	☐	☐	☐	☐	☐
I think that *Journalen* is easy to use	☐	☐	☐	☐	☐
I think I would need personal technical support to use *Journalen*	☐	☐	☐	☐	☐
I think that the various functions in *Journalen* work well together	☐	☐	☐	☐	☐
I think there are many elements in *Journalen* that are not consistent	☐	☐	☐	☐	☐
I think that most people could learn to use *Journalen* fairly quickly	☐	☐	☐	☐	☐
I think that *Journalen* is difficult to use	☐	☐	☐	☐	☐
I feel very safe and secure (about what I do) when I use *Journalen*	☐	☐	☐	☐	☐
I need to learn quite a lot before I can start using *Journalen*	☐	☐	☐	☐	☐
I as a user of 1177.se find it difficult to find the link to *Journalen*	☐	☐	☐	☐	☐

Other (specify): ____

INFORMATION CONTENT

9. How accurate are the following statements regarding your understanding of the content in *Journalen*?

	Strongly Disagree	*Disagree*	*Neutral*	*Agree*	*Strongly Agree*
I understand most of what is in the medical records	☐	☐	☐	☐	☐
I think that the medical records contain too much technical language	☐	☐	☐	☐	☐
I think that the medical records should be written more comprehensible	☐	☐	☐	☐	☐
I understand most of the test results	☐	☐	☐	☐	☐
I understand most of the log list	☐	☐	☐	☐	☐
I understand most of the referral function	☐	☐	☐	☐	☐

10. What would you do if you see something in *Journalen* you do not understand?

	Strongly Disagree	*Disagree*	*Neutral*	*Agree*	*Strongly Agree*
Contact the current healthcare unit via phone	☐	☐	☐	☐	☐
Ask medical staff at the next visit	☐	☐	☐	☐	☐
Ask a medically trained person, e.g. via phone on 1177	☐	☐	☐	☐	☐
Ask an anonymous question via www.1177.se	☐	☐	☐	☐	☐
Ask someone who I know personally, in family or among acquaintances	☐	☐	☐	☐	☐
Look for information myself, e.g. via Internet	☐	☐	☐	☐	☐
Use social media, e.g. discussion forums	☐	☐	☐	☐	☐
Do nothing	☐	☐	☐	☐	☐

Other (specify): ____

11. How do you share information in *Journalen* with others?

	Strongly Disagree	*Disagree*	*Neutral*	*Agree*	*Strongly Agree*
I use the Share-function in *Journalen* to appoint representatives and share information	☐	☐	☐	☐	☐
Discuss it with relative	☐	☐	☐	☐	☐
Discuss it with medical staff	☐	☐	☐	☐	☐

Other (specify): ____

If you use the Share-function in *Journalen*, how do you share information?

I share all information in *Journalen* ☐
I share only selected parts in *Journalen* ☐

12. Have you read anything in *Journalen* that made you feel anxious?

Yes ☐
No ☐

If yes, what did you do?

Contacted healthcare via phone ☐
Waited until the next visit ☐
Contacted a friend with medical background ☐
Contacted patient association / patient committee ☐
Tried to find information about it on the Internet ☐
Done nothing ☐

Other (specify): ____

13. Have you read anything in *Journalen* that has made you feel upset?

Yes ☐
No ☐

If you like, specify why you felt upset: ____________________

If yes, what did you do?

Contacted healthcare via phone ☐
Waited until the next visit ☐
Contacted a friend who works in healthcare ☐
Contacted patient association / patient committee ☐
Wrote about it on social media ☐
Done nothing ☐

Other (specify): ____________________

14. Have you read anything in *Journalen* that was wrong, such as wrong information and/or data?

Yes ☐
No ☐
Do not know ☐

If yes, what did you do?

Contacted healthcare via phone ☐
Waited until the next visit ☐
Contacted a friend with medical background ☐
Contacted patient association / patient committee ☐
Tried to find information about it on the Internet ☐
Done nothing ☐

Other (specify): ____________________

15. How accurate is the content of *Journalen*?

	Strongly Disagree	*Disagree*	*Neutral*	*Agree*	*Strongly Agree*
The content in the record reflects the information I think that healthcare has about me	☐	☐	☐	☐	☐
There is information about me that is missing in the record which I think should be there and that the staff should know	☐	☐	☐	☐	☐

If you consider that there is information missing that should be there, specify what type of information is missing: ____________________

16. How important is *Journalen* to make you feel that you are involved in your own care?

	Strongly Disagree	*Disagree*	*Neutral*	*Agree*	*Strongly Agree*
Information in *Journalen* has helped me in communication with medical staff	☐	☐	☐	☐	☐
Information in *Journalen* had a positive impact on the ability to work together with medical staff making decisions about care and treatment	☐	☐	☐	☐	☐
Information in *Journalen* had a positive impact on the ability to follow the prescription of treatment	☐	☐	☐	☐	☐
Information in *Journalen* had a positive impact on the ability to take own steps to improve health	☐	☐	☐	☐	☐

Other (specify): ______________________________

17. How important is it for you to have access to the following information which is wholly or partly based on information contained in *Journalen*?

	Strongly Disagree	*Disagree*	*Neutral*	*Agree*	*Strongly Agree*
Referral (content and how it is handled in care)	☐	☐	☐	☐	☐
List of all pharmaceuticals	☐	☐	☐	☐	☐
Overview of all vaccinations	☐	☐	☐	☐	☐
Results of tests	☐	☐	☐	☐	☐
Overview of all healthcare contacts	☐	☐	☐	☐	☐
Being able to read record entries from psychiatry	☐	☐	☐	☐	☐
Being able to read all types of record entries	☐	☐	☐	☐	☐
Ability to order and manage medical certificate and other certificates	☐	☐	☐	☐	☐
Ability to point out errors I find in the record	☐	☐	☐	☐	☐
Ability to write own comments to text in *Journalen*	☐	☐	☐	☐	☐
Contribute with information on health, e.g. by providing Health declaration for next visit	☐	☐	☐	☐	☐
Contribute with information of self-testing or monitoring at home	☐	☐	☐	☐	☐
Contribute information about expectations for the healthcare visit	☐	☐	☐	☐	☐
Ability to contact healthcare electronically and ask questions about medical record	☐	☐	☐	☐	☐
Ability to communicate electronically with other patients	☐	☐	☐	☐	☐
Ability to block certain medical records from access by other medical staff	☐	☐	☐	☐	☐
See which care units and staff groups have been inside *Journalen* (see log data)	☐	☐	☐	☐	☐
Ability to access information and manage services for my children	☐	☐	☐	☐	☐

Other (specify): ____________________

SECURITY

18. How do you perceive the level of security in *Journalen*?

	Strongly Disagree	*Disagree*	*Neutral*	*Agree*	*Strongly Agree*
As far as I can judge, I think that *Journalen* generally maintains a high level of security.	☐	☐	☐	☐	☐
I trust that only authorized medical staff is accessing my medical records in *Journalen*.	☐	☐	☐	☐	☐
It is good that I as a patient am able to take part in the log list and see who has accessed my patient [illegible]	☐	☐	☐	☐	☐

Other (specify): ____________________

GENERAL QUESTIONS ON INFORMATION

19. How do you receive bad news about your health from your healthcare provider?

By Phone ☐
In connection with a healthcare visit ☐
Via mail ☐
Via other “web / eServices” ☐

Other (specify): ____________________

20. How would you want to receive bad news about your health?

By Phone ☐
In connection with a healthcare visit ☐
Via mail ☐
In connection with that I read alone about it in *Journalen* ☐

Other (specify): ____________________

21. How accurate are the following statements regarding access to health information?

	Strongly Disagree	*Disagree*	*Neutral*	*Agree*	*Strongly Agree*
It is important (for me) to get information about health	☐	☐	☐	☐	☐
I want to get health information from many different sources	☐	☐	☐	☐	☐
I compare the health information I have received from various sources	☐	☐	☐	☐	☐
It is easy to determine in what situations I need health information	☐	☐	☐	☐	☐
I know where I can get health information	☐	☐	☐	☐	☐
I can use the health information I have received or acquired in order to take care of my own or other people's health	☐	☐	☐	☐	☐
I easily get hold of the health information I need					
a) in printed sources (magazines, books)	☐	☐	☐	☐	☐
b) on the internet	☐	☐	☐	☐	☐
c) on TV or radio	☐	☐	☐	☐	☐
d) from medical staff (e.g. doctors, nurses)	☐	☐	☐	☐	☐
e) from other people	☐	☐	☐	☐	☐
f) from healthcare information system etc.	☐	☐	☐	☐	☐
It is difficult to know whom to trust regarding health issues / questions	☐	☐	☐	☐	☐
It is easy to determine whether the health information I received from the following sources are reliable or not:					
a) in printed sources (magazines, books)	☐	☐	☐	☐	☐
b) on the internet	☐	☐	☐	☐	☐
c) on TV or radio	☐	☐	☐	☐	☐
d) from medical staff (e.g., doctors, nurses)	☐	☐	☐	☐	☐
e) from other people	☐	☐	☐	☐	☐
It is difficult to understand words or sentences used in health information	☐	☐	☐	☐	☐
It is easy to understand the drugs package inserts, labels, or prescription	☐	☐	☐	☐	☐
I get way too much information about health	☐	☐	☐	☐	☐
I do not want to think about matters related to health	☐	☐	☐	☐	☐

	Strongly Disagree	*Disagree*	*Neutral*	*Agree*	*Strongly Agree*
I have avoided to read, listen to or watch health information					
a) in printed sources (magazines, books)	☐	☐	☐	☐	☐

b) on the internet	☐	☐	☐	☐	☐
c) on TV or radio	☐	☐	☐	☐	☐
d) from medical staff (e.g. doctors, nurses)	☐	☐	☐	☐	☐
e) from other people	☐	☐	☐	☐	☐
The health information I receive					
a) helps me often to understand or learn something new	☐	☐	☐	☐	☐
b) often confirms my previous understanding	☐	☐	☐	☐	☐
c) often conflict with my previous views	☐	☐	☐	☐	☐
I can affect my own health	☐	☐	☐	☐	☐

22. What other sources do you use to find health information?

www.1177.se ☐
Internetmedicin.se ☐
Netdoktor.se ☐
Health magazines ☐
Books ☐
Peer-to-peer sources (e.g., discussion forums, Facebook) ☐

Other (specify): ______________________________

QUESTIONS ABOUT YOUR HEALTH

23. I am part of one of the following disease groupings:

Cancer ☐
Mental health ☐
Diabetic ☐
High blood pressure ☐

Other (specify): ______________________________

24. I consider myself

	Strongly Disagree	*Disagree*	*Neutral*	*Agree*	*Strongly Agree*
My health is very good	☐	☐	☐	☐	☐
I am very worried about my health	☐	☐	☐	☐	☐
I often think about my health	☐	☐	☐	☐	☐

DEMOGRAPHIC QUESTIONS

Age

Year of Birth: ____________________

Gender

Female ☐
Male ☐
Other ☐

Do you work (or have you worked) in healthcare?

Yes ☐
No ☐

Education? (Select the highest education/degree you have received/completed)

No formal education ☐
Compulsory education ☐
(e.g., elementary school, junior secondary school)
High school, less than 3 years ☐
High school, 3 years or more ☐
Higher education, less than 3 years ☐
Higher education, 3 years or more ☐
Doctorate ☐

Other (specify): ____

I belong to the following county council:

Blekinge county council	☐
Dalarna county council	☐
Region Gotland	☐
Region Gävleborg	☐
Region Halland	☐
Region Jämtland/Härjedalen	☐
Region Jönköpings Iän	☐
Kalmar county council	☐
Kronoberg county council	☐
Norrbotten county council	☐
Region Skåne	☐
Stockholm county council	☐
Sörmland county council	☐
Region Uppsala	☐
Värmland county council	☐
Västerbottens county council	☐
Västernorrland county council	☐
Västmanland county council	☐
Region Västra Götaland	☐
Region Örebro	☐
Region Östergötland	☐

I have also received healthcare in another county council:

Blekinge county council	☐
Dalarna county council	☐
Region Gotland	☐
Region Gävleborg	☐
Region Halland	☐
Region Jämtland/Härjedalen	☐
Region Jönköpings Iän	☐
Kalmar county council	☐
Kronoberg county council	☐
Norrbotten county council	☐
Region Skåne	☐
Stockholm county council	☐
Sörmland county council	☐
Region Uppsala	☐
Värmland county council	☐
Västerbottens county council	☐
Västernorrland county council	☐
Västmanland county council	☐
Region Västra Götaland	☐
Region Örebro	☐
Region Östergötland	☐
