# Supplementary material for: Patients’ Experiences of Accessing Their Electronic Health Records: National Patient Survey in Sweden
Source: J Med Internet Res. 2018 Nov 1;20(11):e278. doi: 10.2196/jmir.9492 (PMC6238103; doi:10.2196/jmir.9492)
Supplement: Multimedia Appendix 2 [file jmir_v20i11e278_app2.pdf]

This appendix provides details on the chosen items from survey questions 3, 4, 5 and 17. On pages 1-10, the cell with the highest percentage value in each column has been shaded. On pages 11-13, answers from current and former healthcare professionals and answers from all other respondents are compared in tables. See questionnaire in Multimedia Appendix 1 for the exact formulations.

Results from question 3 ("I believe that access to medical records online is generally a good reform") from the respective county council

County council	Strongly agree	Agree	Neutral	Disagree	Strongly disagree
Skåne	570/653 (87.3%)	66/653 (10.1%)	6/653 (0.9%)	7/653 (1.1%)	4/653 (0.6%)
Uppsala	417/491 (84.9%)	60/491 (12.2%)	6/491 (1.2%)	2/491 (0.4%)	6/491 (1.2%)
Östergötland	285/341 (83.6%)	42/341 (12.3%)	6/341 (1.8%)	4/341 (1.2%)	4/341 (1.2%)
Västra götaland	250/304 (82.2%)	36/304 (11.8%)	8/304 (2.6%)	8/304 (2.6%)	2/304 (0.7%)
Stockholm	248/291 (85.2%)	30/291 (10.3%)	5/291 (1.7%)	4/291 (1.4%)	4/291 (1.4%)
Jönköping	168/203 (82.8%)	31/203 (15.3%)	2/203 (1.0%)	2/203 (1.0%)	0/203 (0.0%)
Örebro	141/169 (83.4%)	21/169 (12.4%)	6/169 (3.6%)	1/169 (0.6%)	0/169 (0.0%)
Värmland	140/170 (82.4%)	23/170 (13.5%)	4/170 (2.4%)	2/170 (1.2%)	1/170 (0.6%)
Västmanland	130/153 (85.0%)	18/153 (11.8%)	3/153 (2.0%)	0/153 (0.0%)	2/153 (1.3%)
Dalarna	130/153 (85.0%)	20/153 (13.1%)	2/153 (1.3%)	1/153 (0.7%)	0/153 (0.0%)
Sörmland	114/138 (82.6%)	20/138 (14.5%)	2/138 (1.4%)	0/138 (0.0%)	2/138 (1.4%)
Västerbotten	111/139 (80.0%)	21/139 (15.1%)	2/139 (1.4%)	3/139 (2.2%)	2/139 (2.2%)
Kronoberg	110/128 (85.9%)	13/128 (10.2%)	2/128 (1.6%)	2/128 (1.6%)	1/128 (0.8%)
Kalmar	102/128 (79.7%)	21/128 (16.4%)	2/128 (1.6%)	3/128 (2.3%)	0/128 (0.0%)
Blekinge	86/101 (85.1%)	10/101 (9.9%)	3/101 (3.0%)	0/101 (0.0%)	2/101 (2.0%)
Norrbotten	79/94 (84.0%)	9/94 (9.6%)	3/94 (3.2%)	1/94 (1.1%)	2/94 (2.1%)

Results from question 4 ("Why do you use "Journalen"?")

Reason for use	Strongly agree	Agree	Neutral	Disagree	Strongly disagree
General interest	803/2426 (33.1%)	751/2426 (31.0%)	449/2426 (18.5%)	184/2426 (7.6%)	239/2426 (9.9%)
Own overview	1913/2514 (76.1%)	419/2514 (16.7%)	99/2514 (3.9%)	29/2514 (1.2%)	54/2514 (2.1%)
Overview relative	336/2390 (14.1%)	204/2390 (8.5%)	342/2390 (14.3%)	171/2390 (7.2%)	1337/2390 (55.9%)
Insecure about care accuracy	295/2427 (12.2%)	437/2427 (18.0%)	564/2427 (23.2%)	426/2427 (17.6%)	705/2427 (29.0%)
Follow-up on visit	1551/2512 (61.7%)	670/2512 (26.7%)	146/2512 (5.8%)	45/2512 (1.8%)	100/2512 (4.0%)
Suspect error	205/2432 (8.4%)	308/2432 (12.7%)	534/2432 (22.0%)	485/2432 (19.9%)	900/2432 (37.0%)
Prepare for visit	675/2457 (27.5%)	727/2457 (29.6%)	504/2457 (20.5%)	221/2457 (9.0%)	330/2457 (13.4%)
More involved	1372/2470 (55.5%)	651/2470 (26.4%)	283/2470 (11.5%)	57/2470 (2.3%)	107/2470 (4.3%)

Results from question 5 ("How important is it for you to be able to access patient information?")

Alternative	Strongly agree	Agree	Neutral	Disagree	Strongly disagree
Improves communication	1418/2525 (56.2%)	705/2525 (27.9%)	280/2525 (11.1%)	67/2525 (2.7%)	55/2525 (2.2%)
Better care	1082/2486 (43.5%)	744/2486 (29.9%)	504/2486 (20.3%)	85/2486 (3.4%)	71/2486 (2.9%)
Better understanding	1305/2494 (52.3%)	698/2494 (28.0%)	343/2494 (13.8%)	83/2494 (3.3%)	65/2494 (2.6%)
Feel safe	1261/2502 (50.4%)	733/2502 (29.3%)	379/2502 (15.1%)	62/2502 (2.5%)	67/2502 (2.7%)
Feel informed	1792/2506 (71.5%)	556/2506 (22.2%)	105/2506 (4.2%)	20/2506 (0.8%)	33/2506 (1.3%)
Better self care	589/2477 (23.8%)	618/2477 (24.9%)	928/2477 (37.5%)	158/2477 (6.4%)	184/2477 (7.4%)
Better care of relative	266/2401 (11.1%)	231/2401 (9.6%)	842/2401 (35.1%)	146/2401 (6.1%)	916/2401 (38.2%)
Shared decision	761/2464 (30.9%)	531/2464 (21.6%)	625/2464 (25.4%)	122/2464 (5.0%)	425/2464 (17.2%)
Own documentation	1104/2451 (45.0%)	651/2451 (26.6%)	380/2451 (15.5%)	111/2451 (4.5%)	205/2451 (8.4%)
No relevance	50/2346 (2.1%)	47/2346 (2.0%)	299/2346 (12.7%)	222/2346 (9.5%)	1728/2346 (73.7%)

Results from question 17 (“How important is it for you to have access the following information which is wholly or partly based on information contained in “Journalen” “) from the respective county councils

Entire nation

Information	Strongly agree	Agree	Neutral	Disagree	Strongly disagree
Referrals	1775/2444 (72.6%)	425/2444 (17.4%)	199/2444 (8.1%)	18/2444 (0.7%)	29/2444 (1.2%)
Med. list	1864/2454 (76.0%)	333/2454 (13.6%)	186/2454 (7.6%)	32/2454 (1.3%)	39/2454 (1.6%)
Immunizations	1570/2434 (64.5%)	387/2434 (15.9%)	366/2434 (15.0%)	41/2434 (1.7%)	70/2434 (2.9%)
Test results	2049/2455 (83.5%)	283/2455 (11.5%)	84/2455 (3.4%)	11/2455 (0.4%)	28/2455 (1.1%)
Visit history	1802/2437 (73.9%)	407/2437 (16.7%)	172/2437 (7.1%)	25/2437 (1.0%)	31/2437 (1.3%)
Log list	1314/2416 (54.4%)	482/2416 (20.0%)	353/2416 (14.6%)	79/2416 (3.3%)	188/2416 (7.8%)
Health declarations	1168/2421 (48.2%)	633/2421 (26.1%)	453/2421 (18.7%)	66/2421 (2.7%)	101/2421 (4.2%)
Notes	1920/2453 (78.3%)	314/2453 (12.8%)	141/2453 (5.7%)	35/2453 (1.4%)	43/2453 (1.8%)

Region Skåne

Information	Strongly agree	Agree	Neutral	Disagree	Strongly disagree
Referrals	486/651 (74.7%)	101/651 (15.5%)	51/651 (7.8%)	6/651 (0.9%)	7/651 (1.1%)
Med. list	505/649 (77.8%)	77/649 (11.9%)	51/649 (7.9%)	9/649 (1.4%)	7/649 (1.1%)

Immunizations	435/644 (67.5%)	98/644 (15.2%)	84/644 (13.0%)	8/644 (1.2%)	19/644 (3.0%)
Test results	547/648 (84.4%)	59/648 (9.1%)	27/648 (4.2%)	6/648 (0.9%)	9/648 (1.4%)
Visit history	506/648 (78.1%)	84/648 (13.0%)	45/648 (6.9%)	7/648 (1.1%)	6/648 (0.9%)
Log list	356/642 (55.5%)	118/642 (18.4%)	97/642 (15.1%)	24/642 (3.7%)	47/642 (7.3%)
Health declarations	319/646 (49.4%)	155/646 (24.0%)	131/646 (20.3%)	16/646 (2.5%)	25/646 (3.9%)
Notes	537/650 (82.6%)	66/650 (10.2%)	25/650 (3.8%)	9/650 (1.4%)	13/650 (2.0%)

Region Uppsala

**Information**	**Strongly agree**	**Agree**	**Neutral**	**Disagree**	**Strongly disagree**
Referrals	352/486 (72.4%)	99/486 (20.4%)	29/486 (6.0%)	3/486 (0.6%)	3/486 (0.6%)
Med. list	354/488 (72.5%)	79/488 (16.2%)	45/488 (9.2%)	1/488 (0.2%)	9/488 (1.8%)
Immunizations	302/485 (62.3%)	78/485 (16.1%)	81/485 (16.7%)	9/485 (1.9%)	15/485 (3.1%)
Test results	410/491 (83.5%)	66/491 (13.4%)	10/491 (2.0%)	0/491 (0.0%)	5/491 (1.0%)
Visit history	349/484 (72.1%)	87/484 (18.0%)	36/484 (7.4%)	5/484 (1.0%)	7/484 (1.4%)
Log list	274/484 (56.6%)	94/484 (19.4%)	69/484 (14.3%)	13/484 (2.7%)	34/484 (7.0%)
Health declarations	236/482 (49.0%)	115/482 (23.9%)	99/482 (20.5%)	16/482 (3.3%)	16/482 (3.3%)
Notes	383/487 (78.6%)	65/487 (13.3%)	31/487 (6.4%)	4/487 (0.8%)	4/487 (0.8%)

Region Östergötland

Information	Strongly agree	Agree	Neutral	Disagree	Strongly disagree
Referrals	247/338 (73.1%)	55/338 (16.3%)	28/338 (8.3%)	4/338 (1.2%)	4/338 (1.2%)
Med. list	253/338 (74.9%)	54/338 (16.0%)	24/338 (7.1%)	3/338 (0.9%)	4/338 (1.2%)
Immunizations	220/334 (65.9%)	55/334 (16.5%)	46/334 (13.8%)	8/334 (2.4%)	5/334 (1.5%)
Test results	284/338 (84.0%)	41/338 (12.1%)	8/338 (2.4%)	2/338 (0.6%)	3/338 (0.9%)
Visit history	253/335 (75.5%)	57/335 (17.0%)	17/335 (5.1%)	5/335 (1.5%)	3/335 (0.9%)
Log list	179/331 (54.1%)	75/331 (22.7%)	46/331 (13.9%)	11/331 (3.3%)	20/331 (6.0%)
Health declarations	156/330 (47.3%)	99/330 (30.0%)	54/330 (16.4%)	9/330 (2.7%)	12/330 (3.6%)
Notes	253/338 (74.9%)	54/338 (16.0%)	22/338 (6.5%)	5/338 (1.5%)	4/338 (1.2%)

Region Västra Götaland

Information	Strongly agree	Agree	Neutral	Disagree	Strongly disagree
Referrals	229/305 (75.1%)	51/305 (16.7%)	15/305 (4.9%)	3/305 (1.0%)	7/305 (2.3%)
Med. list	236/307 (76.9%)	35/307 (11.4%)	26/307 (8.5%)	4/307 (1.3%)	6/307 (2.0%)
Immunizations	197/302 (65.2%)	54/302 (17.9%)	37/302 (12.3%)	4/302 (1.3%)	10/302 (3.3%)
Test results	258/309 (83.5%)	35/309 (11.3%)	9/309 (2.9%)	3/309 (1.0%)	4/309 (1.3%)
Visit history	224/305 (73.4%)	47/305 (15.4%)	23/305 (7.5%)	5/305 (1.6%)	6/305 (2.0%)
Log list	151/297 (50.8%)	61/297 (20.5%)	39/297 (13.1%)	13/297 (4.4%)	33/297 (11.1%)
Health declarations	157/301 (52.2%)	69/301 (22.9%)	51/301 (16.9%)	12/301 (4.0%)	12/301 (4.0%)
Notes	236/307 (76.9%)	38/307 (12.4%)	20/307 (6.5%)	8/307 (2.6%)	5/307 (1.6%)

Region Jönköping

Information	Strongly agree	Agree	Neutral	Disagree	Strongly disagree
Referrals	134/200 (67.0%)	37/200 (18.5%)	23/200 (11.5%)	2/200 (1.0%)	4/200 (2.0%)
Med. list	152/200 (76.0%)	27/200 (13.5%)	17/200 (8.5%)	3/200 (1.5%)	1/200 (0.5%)
Immunizations	131/200 (65.5%)	40/200 (20.0%)	21/200 (10.5%)	4/200 (2.0%)	4/200 (2.0%)
Test results	166/202 (82.2%)	26/202 (12.9%)	8/202 (4.0%)	0/202 (0.0%)	2/202 (1.0%)
Visit history	139/198 (70.2%)	46/198 (23.2%)	8/198 (4.0%)	3/198 (1.5%)	2/198 (1.0%)
Log list	118/198 (60.0%)	38/198 (19.2%)	21/198 (10.6%)	7/198 (3.5%)	14/198 (7.1%)
Health declarations	101/199 (50.8%)	58/199 (29.1%)	32/199 (16.1%)	4/199 (2.0%)	4/199 (2.0%)
Notes	144/200 (72.0%)	38/200 (19.0%)	12/200 (6.0%)	0/200 (0.0%)	6/200 (3.0%)

Region Örebro

Information	Strongly agree	Agree	Neutral	Disagree	Strongly disagree
Referrals	111/166 (66.9%)	29/166 (17.5%)	20/166 (12.0%)	1/166 (0.6%)	5/166 (3.0%)
Med. list	130/167 (77.8%)	23/167 (13.8%)	11/167 (6.6%)	1/167 (0.6%)	2/167 (1.2%)
Immunizations	97/167 (58.1%)	31/167 (18.6%)	32/167 (19.2%)	2/167 (1.2%)	5/167 (3.0%)
Test results	135/169 (79.9%)	23/169 (13.6%)	9/169 (5.3%)	0/169 (0.0%)	2/169 (1.2%)
Visit history	122/166 (73.5%)	26/166 (15.7%)	13/166 (7.8%)	0/166 (0.0%)	5/166 (3.0%)
Log list	93/169 (55.0%)	30/169 (17.8%)	27/169 (16.0%)	8/169 (4.7%)	11/169 (6.5%)
Health declarations	77/166 (46.4%)	50/166 (30.1%)	26/166 (15.7%)	5/166 (3.0%)	8/166 (4.8%)
Notes	132/168 (78.6%)	20/168 (11.9%)	14/168 (8.3%)	2/168 (1.2%)	0/168 (0.0%)

Värmland county council

Information	Strongly agree	Agree	Neutral	Disagree	Strongly disagree
Referrals	110/170 (64.7%)	36/170 (21.2%)	16/170 (9.4%)	4/170 (2.4%)	4/170 (2.4%)
Med. list	122/169 (72.2%)	24/169 (14.2%)	14/169 (8.3%)	7/169 (4.1%)	2/169 (1.2%)
Immunizations	97/168 (57.7%)	31/168 (18.5%)	31/168 (18.5%)	6/168 (3.6%)	3/168 (1.8%)
Test results	127/170 (74.7%)	34/170 (20.0%)	6/170 (3.5%)	1/170 (0.6%)	2/170 (1.2%)
Visit history	100/169 (59.2%)	41/169 (24.3%)	19/169 (11.2%)	4/169 (2.4%)	5/169 (3.0%)
Log list	85/165 (51.5%)	31/165 (18.8%)	22/167 (13.2%)	7/165 (4.2%)	20/165 (12.1%)
Health declarations	75/167 (44.9%)	43/167 (25.7%)	37/167 (22.2%)	1/167 (0.6%)	11/167 (6.6%)
Notes	123/168 (73.2%)	28/168 (16.7%)	10/168 (6.0%)	6/168 (3.6%)	1/168 (0.6%)

Västmanland county council

Information	Strongly agree	Agree	Neutral	Disagree	Strongly disagree
Referrals	121/156 (77.6%)	21/156 (13.5%)	12/156 (7.7%)	0/156 (0.0%)	2/156 (1.3%)
Med. list	122/155 (78.7%)	17/155 (11.0%)	10/155 (6.5%)	1/155 (0.6%)	5/155 (3.2%)
Immunizations	100/155 (64.5%)	11/155 (7.1%)	31/155 (20.0%)	2/155 (1.3%)	11/155 (7.1%)
Test results	146/157 (93.0%)	5/157 (3.2%)	3/157 (1.9%)	0/157 (0.0%)	3/157 (1.9%)
Visit history	125/154 (81.2%)	16/154 (10.4%)	10/154 (6.5%)	0/154 (0.0%)	3/154 (1.9%)
Log list	100/155 (64.5%)	29/155 (18.7%)	16/155 (10.3%)	2/155 (1.3%)	8/155 (5.2%)
Health declarations	74/154 (48.1%)	48/154 (31.2%)	23/154 (14.9%)	2/154 (1.3%)	7/154 (4.5%)
Notes	127/156 (81.4%)	16/156 (10.3%)	6/156 (3.8%)	2/156 (1.3%)	5/156 (3.2%)

Dalarna county council

Information	Strongly agree	Agree	Neutral	Disagree	Strongly disagree
Referrals	108/152 (71.1%)	24/152 (15.8%)	18/152 (11.8%)	1/152 (0.7%)	1/152 (0.7%)
Med. list	104/152 (68.4%)	27/152 (17.8%)	18/152 (11.8%)	2/152 (1.3%)	1/152 (0.7%)
Immunizations	85/151 (56.3%)	28/151 (18.5%)	26/151 (17.2%)	5/151 (3.3%)	7/151 (4.6%)
Test results	120/151 (79.5%)	22/151 (14.6%)	6/151 (4.0%)	0/151 (0.0%)	3/151 (2.0%)
Visit history	118/152 (77.6%)	21/152 (13.8%)	9/152 (5.9%)	2/152 (1.3%)	2/152 (1.3%)
Log list	80/151 (53.0%)	28/151 (18.5%)	22/151 (14.6%)	7/151 (4.6%)	14/151 (9.3%)
Health declarations	77/152 (50.7%)	43/152 (28.3%)	19/152 (12.5%)	4/152 (2.6%)	9/152 (5.9%)
Notes	125/152 (82.2%)	14/152 (9.2%)	8/152 (5.3%)	2/152 (1.3%)	3/152 (2.0%)

Sörmland county council

Information	Strongly agree	Agree	Neutral	Disagree	Strongly disagree
Referrals	98/136 (72.1%)	23/136 (16.9%)	14/136 (10.3%)	0/136 (0.0%)	3/136 (2.2%)
Med. list	107/136 (78.7%)	14/136 (10.3%)	12/136 (8.8%)	0/136 (0.0%)	3/136 (2.2%)
Immunizations	85/135 (63.0%)	12/135 (8.9%)	33/135 (24.4%)	0/135 (0.0%)	5/135 (3.7%)
Test results	114/135 (84.4%)	12/135 (8.9%)	7/135 (5.2%)	0/135 (0.0%)	2/135 (1.5%)
Visit history	101/135 (74.8%)	18/135 (13.3%)	11/135 (8.1%)	1/135 (0.7%)	4/135 (3.0%)
Log list	66/135 (48.9%)	35/135 (25.9%)	18/135 (13.3%)	4/135 (3.0%)	12/135 (8.9%)
Health declarations	64/133 (48.1%)	35/133 (26.3%)	24/133 (18.0%)	5/133 (3.8%)	5/133 (3.8%)
Notes	111/137 (81.0%)	16/137 (11.7%)	6/137 (4.4%)	1/137 (0.7%)	3/137 (2.2%)

Västerbotten county council

Information	Strongly agree	Agree	Neutral	Disagree	Strongly disagree
Referrals	90/136 (66.2%)	26/136 (19.1%)	19/136 (14.0%)	1/136 (0.7%)	0/136 (0.0%)
Med. list	106/135 (78.5%)	17/135 (12.6%)	11/135 (8.1%)	0/135 (0.0%)	1/135 (0.7%)
Immunizations	94/135 (69.6%)	21/135 (15.6%)	19/135 (14.1%)	1/135 (0.7%)	1/135 (0.0%)
Test results	107/135 (79.3%)	19/135 (14.1%)	8/135 (5.9%)	1/135 (0.7%)	0/135 (0.0%)
Visit history	99/136 (72.8%)	24/136 (17.6%)	11/136 (8.1%)	2/136 (1.5%)	0/136 (0.0%)
Log list	69/135 (51.1%)	25/135 (18.5%)	26/135 (19.3%)	7/135 (5.2%)	8/135 (5.9%)
Health declarations	66/135 (48.9%)	41/135 (30.4%)	21/135 (15.6%)	2/135 (1.5%)	5/135 (3.7%)
Notes	98/137 (71.5%)	17/137 (12.4%)	15/137 (10.9%)	5/137 (3.6%)	2/137 (1.5%)

Kronoberg county council

Information	Strongly agree	Agree	Neutral	Disagree	Strongly disagree
Referrals	91/125 (72.8%)	20/125 (16.0%)	13/125 (10.4%)	1/125 (0.8%)	0/125 (0.0%)
Med. list	98/123 (79.7%)	13/123 (10.6%)	12/123 (9.8%)	0/123 (0.0%)	0/123 (0.0%)
Immunizations	86/125 (68.8%)	15/125 (12.0%)	19/125 (15.2%)	2/125 (1.6%)	3/125 (2.4%)
Test results	105/126 (83.3%)	14/126 (11.1%)	6/126 (4.8%)	0/126 (0.0%)	1/126 (0.8%)
Visit history	89/125 (71.2%)	27/125 (21.6%)	9/125 (7.2%)	0/125 (0.0%)	0/125 (0.0%)
Log list	71/127 (55.9%)	23/127 (18.1%)	18/127 (14.2%)	2/127 (1.6%)	13/127 (10.2%)
Health declarations	65/125 (52.0%)	33/125 (26.4%)	20/125 (16.0%)	2/125 (1.6%)	5/125 (4.0%)
Notes	96/126 (76.2%)	16/126 (12.7%)	12/126 (9.5%)	1/126 (0.8%)	1/126 (0.8%)

Kalmar county council

Information	Strongly agree	Agree	Neutral	Disagree	Strongly disagree
Referrals	93/125 (74.4%)	20/125 (16.0%)	10/125 (8.0%)	0/125 (0.0%)	2/125 (1.6%)
Med. list	102/127 (80.3%)	15/127 (11.8%)	6/127 (4.7%)	3/127 (2.4%)	1/127 (0.8%)
Immunizations	88/124 (71.0%)	12/124 (9.7%)	18/124 (14.5%)	2/124 (1.6%)	4/124 (3.2%)
Test results	106/126 (84.1%)	13/126 (10.3%)	6/126 (4.8%)	0/126 (0.0%)	1/126 (0.8%)
Visit history	100/127 (78.7%)	19/127 (15.0%)	5/127 (3.9%)	1/127 (0.8%)	2/127 (1.6%)
Log list	70/123 (56.9%)	31/123 (25.2%)	13/123 (10.6%)	1/123 (0.8%)	8/123 (6.5%)
Health declarations	69/122 (56.6%)	29/122 (23.8%)	15/122 (12.3%)	1/122 (0.8%)	8/122 (6.6%)
Notes	95/126 (75.4%)	21/126 (16.7%)	6/126 (4.8%)	2/126 (1.6%)	2/126 (1.6%)

Region Halland

Information	Strongly agree	Agree	Neutral	Disagree	Strongly disagree
Referrals	73/100 (73.0%)	14/100 (14.0%)	9/100 (9.0%)	0/100 (0.0%)	4/100 (4.0%)
Med. list	83/101 (82.2%)	9/101 (8.9%)	6/101 (5.9%)	1/101 (1.0%)	2/101 (2.0%)
Immunizations	68/100 (68.0%)	12/100 (12.0%)	14/100 (14.0%)	2/100 (2.0%)	4/100 (4.0%)
Test results	83/101 (82.2%)	9/101 (8.9%)	4/101 (4.0%)	2/101 (2.0%)	3/101 (3.0%)
Visit history	75/99 (75.8%)	13/99 (13.1%)	6/99 (6.1%)	2/99 (2.0%)	3/99 (5.1%)
Log list	54/99 (54.5%)	20/99 (20.2%)	14/99 (14.1%)	3/99 (5.1%)	8/99 (8.1%)
Health declarations	55/99 (55.6%)	20/99 (20.2%)	15/99 (15.2%)	4/99 (4.0%)	5/99 (5.1%)
Notes	77/102 (75.5%)	13/102 (12.7%)	4/102 (3.9%)	6/102 (5.9%)	2/102 (2.0%)

Blekinge county council

Information	Strongly agree	Agree	Neutral	Disagree	Strongly disagree
Referrals	64/92 (69.6%)	16/92 (17.4%)	10/92 (10.9%)	0/92 (0.0%)	2/92 (2.2%)
Med. list	68/91 (74.7%)	13/91 (14.3%)	7/91 (7.7%)	2/91 (2.2%)	1/91 (1.1%)
Immunizations	60/90 (66.7%)	16/90 (17.8%)	11/90 (12.2%)	1/90 (1.1%)	2/90 (2.2%)
Test results	73/90 (81.1%)	11/90 (12.2%)	5/90 (5.6%)	1/90 (1.1%)	0/90 (0.0%)
Visit history	67/92 (72.8%)	19/92 (20.7%)	6/92 (6.5%)	0/92 (0.0%)	0/92 (0.0%)
Log list	49/89 (55.1%)	22/89 (24.7%)	14/89 (15.7%)	0/89 (0.0%)	4/89 (4.5%)
Health declarations	52/91 (57.1%)	24/91 (26.4%)	7/91 (7.7%)	3/91 (3.3%)	4/91 (4.4%)
Notes	68/90 (75.6%)	12/90 (13.3%)	6/90 (6.7%)	2/90 (2.2%)	2/90 (2.2%)

Norrbotten county council

Information	Strongly agree	Agree	Neutral	Disagree	Strongly disagree
Referrals	68/92 (73.9%)	16/92 (17.4%)	5/92 (5.4%)	2/92 (2.2%)	1/92 (1.1%)
Med. list	69/93 (74.2%)	10/93 (10.8%)	7/93 (7.5%)	5/93 (5.4%)	2/93 (2.2%)
Immunizations	56/92 (60.9%)	17/92 (18.5%)	15/92 (16.3%)	3/92 (3.3%)	1/92 (1.1%)
Test results	78/92 (84.8%)	8/92 (8.7%)	5/92 (5.4%)	0/92 (0.0%)	1/92 (1.1%)
Visit history	62/91 (68.1%)	22/91 (24.2%)	5/91 (5.5%)	1/91 (1.1%)	1/91 (1.1%)
Log list	54/92 (58.7%)	15/92 (16.3%)	14/92 (15.2%)	3/92 (3.3%)	6/92 (6.5%)
Health declarations	45/93 (48.4%)	18/93 (19.4%)	18/93 (19.4%)	5/93 (5.4%)	7/93 (7.5%)
Notes	63/93 (67.7%)	17/93 (18.3%)	10/93 (10.8%)	1/93 (1.1%)	2/93 (2.2%)

Comparisons between answers from current and former healthcare professionals and answers from all other respondents (the translation 1=Strongly disagree, 5=Strongly agree has been used in all calculations)

Q3: What is your attitude towards "*Journalen*"?

a. I believe that access to medical records online is generally a good reform
b. I believe that access to "Journalen" is good for me

Question	N	Mean	St. dev.	p-value (95%)
3a-professionals	1018	4.70	0.699	P<0.001
3a-others	1395	4.85	0.515	
3b-professionals	1015	4.82	0.560	P=0.005
3b-others	1385	4.87	0.496	

Q4: Why do you use "*Journalen*"?

a. Mostly general interest
b. To get an overview of my medical history and treatment
c. To get an overview of my relatives' medical history and treatment
d. Because I am not sure if I got the right care
e. To follow up what has been said during a health care visit
f. Because I suspect inaccuracies
g. To prepare for my health care visit
h. To become more involved in my care

Question	N	Mean	St. dev.	p-value (95%)
4a-professionals	990	3.74	1.257	P=0.199
4a-others	1316	3.68	1.275	
4b-professionals	1013	4.59	0.854	P=0.128
4b-others	1379	4.65	0.764	
4c-professionals	974	2.20	1.534	P=0.976
4c-others	1301	2.18	1.497	
4d-professionals	985	2.54	1.356	P=0.001
4d-others	1323	2.74	1.384	
4e-professionals	1013	4.39	0.992	P=0.829
4e-others	1375	4.41	0.955	
4f-professionals	983	2.32	1.313	P=0.497
4f-others	1329	2.35	1.300	
4g-professionals	987	3.38	1.391	P=0.003
4g-others	1354	3.57	1.295	
4h-professionals	1004	4.28	1.058	P=0.161
4h-others	1347	4.25	1.029	

Q5: How important is it for you to be able to access patient information?

a. It improves communication between medical staff and me
b. It leads to improvements in health and social care
c. It improves the understanding of the condition
d. It makes me feel safe
e. It makes me feel informed
f. It leads to that I can take care of my health better
g. It leads to that I can take care of my relatives health better
h. It is essential that I am able to actively participate in decisions about my or my relatives' health
i. For my own documentation
j. It has no relevance

Question	N	Mean	St. dev.	p-value (95%)
5a-professionals	1015	4.24	1.003	P<0.001
5a-others	1391	4.41	0.849	
5b-professionals	1005	4.01	1.071	P=0.013
5b-others	1366	4.14	0.952	
5c-professionals	1001	4.14	1.057	P<0.001
5c-others	1374	4.33	0.899	
5d-professionals	1009	4.22	0.985	P=0.852
5d-others	1373	4.24	0.944	
5e-professionals	1010	4.57	0.799	P=0.021
5e-others	1376	4.67	0.621	
5f-professionals	1001	3.42	1.179	P<0.001
5f-others	1356	3.60	1.095	
5g-professionals	970	2.51	1.393	P=0.913
5g-others	1320	2.49	1.348	
5h-professionals	997	3.40	1.444	P=0.377
5h-others	1348	3.47	1.388	
5i-professionals	994	3.77	1.321	P<0.001
5i-others	1346	4.09	1.158	
5j-professionals	951	1.54	0.958	P=0.021
5j-others	1287	1.46	0.915	

Q17: How important is it for you to have access the following information which is wholly or partly based on information contained in “*Journalen*”

a. Referral (content and how it is handled in care)
b. List of all pharmaceuticals
c. Overview of all vaccinations
d. Results of tests
e. Overview of all health care contacts
f. Being able to read all types of record entries
g. Contribute with information on health, for example by providing health declaration for next visit
h. See which care units and staff groups have been inside “*Journalen*” (see log data)

**Question**	**N**	**Mean**	**St. dev.**	**p-value (95%)**
17a-professionals	1018	4.59	0.776	P=0.914
17a-others	1375	4.59	0.771	
17b-professionals	1015	4.62	0.822	P=0.368
17b-others	1384	4.60	0.803	
17c-professionals	1010	4.44	0.987	P<0.001
17c-others	1371	4.33	0.987	
17d-professionals	1020	4.77	0.623	P=0.612
17d-others	1383	4.75	0.654	
17e-professionals	1017	4.60	0.792	P=0.701
17e-others	1366	4.62	0.752	
17f-professionals	1022	4.62	0.885	P=0.547
17f-others	1377	4.66	0.729	
17g-professionals	1014	4.22	1.051	P<0.001
17g-others	1354	4.05	1.075	
17h-professionals	1008	4.16	1.226	P=0.004
17h-others	1355	4.05	1.231	
